# Effect of Carbon on Dislocation Loops Formation during Self-Ion Irradiation in Fe-Cr Alloys at High Temperatures

**DOI:** 10.3390/ma15062211

**Published:** 2022-03-17

**Authors:** Tiantian Shi, Wenbo Liu, Zhengxiong Su, Xu Yan, Chenyang Lu, Di Yun

**Affiliations:** Department of Nuclear Science and Technology, Xi’an Jiaotong University, Xi’an 710049, China; sttian@stu.xjtu.edu.cn (T.S.); liuwenbo@xjtu.edu.cn (W.L.); suzhengxiong@stu.xjtu.edu.cn (Z.S.); yanxu123@stu.xjtu.edu.cn (X.Y.); chenylu@xjtu.edu.cn (C.L.)

**Keywords:** dislocation loops, self-ion irradiation, Fe-Cr alloys, carbon

## Abstract

In this study, two types of ferritic model alloys (Fe-9Cr and Fe-9Cr-C) were simultaneously irradiated with 3.5 MeV Fe^13+^ ions at 450 °C and 550 °C to a dose of 3dpa at the peak damage region, respectively. Transmission electron microscopy (TEM) was used to investigate the microstructural evolution of the Fe-Cr alloys after irradiation. The experimental results showed that the size of the dislocation loops formed in the Fe-9Cr-C alloy was larger than that in the Fe-9Cr alloy, but the loop density of the Fe-9Cr-C alloy was lower than that of the Fe-9Cr alloy after irradiation at 450 °C. The reason for this phenomenon was attributed to the fact that loops formed in Fe-9Cr-C alloy have greater capture efficiency for interstitial atoms. Compared to Fe-Cr alloys irradiated at 450 °C, high-density loops were not observed in the Fe-Cr alloys irradiated at 550 °C; the number of dislocation loops in the Fe-Cr alloys irradiated at 550 °C significantly decreased due to the rapid conversion of the dislocation loops into network dislocations. In addition, subgrains were observed in the Fe-Cr alloys after irradiation. The underlying reason behind the formation of subgrains is discussed in detail.

## 1. Introduction

Due to the favorable properties of ferritic/martensitic (F/M) steels with Cr content ranging from 9% to 12%, such as higher thermal conductivity, lower thermal expansion [1], and higher swelling resistance compared to austenitic steels [1,2], they are a candidate for the structural materials for the Generation IV fission reactors and fusion reactors [3]. However, hardening and embrittlement induced by low-temperature irradiation (<350 °C) is still a key issue for the application of F/M steels [4]. Extensive research has shown that irradiation leads to the formation of different types of nanofeatures in these steels, such as dislocation loops [5], α’-phase particles [6,7], and G-phase particles [8] et al. These defect clusters can impede dislocation movement, which is regarded as the main reason for the irradiation hardening and embrittlement [1].

In the past several decades, Fe-Cr model steels have been widely used to study the mechanisms of the irradiation-induced microstructure evolution in complex F/M steels. The effects of Cr concentration of Fe-Cr alloys on cavity swelling [9], irradiation-induced segregation [10,11], dislocation loops [12,13,14,15], irradiation-induced precipitation [6,7], and mechanical properties [16,17] have been revealed by extensive experimental investigations. Besides the Cr concentration, the concentration of interstitial elements, such as carbon (C) and nitrogen (N), can also affect the microstructural evolution of the Fe-Cr alloys upon irradiation. Atomistic simulations have revealed that C atoms have different interactions with self-interstitial atoms (SIAs), vacancies, and dislocation loops [18,19,20]. N atoms have similar properties to C atoms in terms of the migration energies as well as interaction with a vacancy or with an SIA [18]. These atomistic simulations indicated that the interstitial elements can affect the microstructural evolution of ferritic steels because of interactions between these interstitial elements and defects. The experiment conducted by Druzhkov et al. [21] by Positron Annihilation Spectroscopy (PAS) also indicated that a larger S-parameter was obtained in Fe-Cr-based alloys with a higher C content irradiated by electrons below 350 °C. This is attributed to the suppression of the mutual recombination of vacancies with SIAs under irradiation since the mobility of vacancies is reduced due to the interaction with C atoms. The experimental research of Konstantinović et al. [22] suggested that C atoms, which dissolved in the matrix, affected the distribution of the dislocation loops in the Fe-Cr alloys irradiated with a neutron at 300 °C. However, there is still a lack of experimental studies about the effects of interstitial elements on the radiation-induced loop microstructure in the Fe-Cr alloys, especially at higher irradiation temperatures.

In the present work, two types of Fe-9Cr model alloys were designed. The aim of this paper is to investigate the effect of interstitial carbon on the microstructural evolution during self-ion radiation in bulk specimens of Fe-9Cr alloys at high temperatures. The specimens of model alloys were irradiated simultaneously with 3.5 MeV Fe^13+^ at 450 °C and 550 °C to a dose of 3dpa at the peak damage region. Transmission electron microscopy (TEM) was used to characterize the microstructures before and after irradiation.

## 2. Materials and Experimental Procedure

### 2.1. Materials

The chemical compositions of the Fe-9Cr alloys used in this work are listed in Table 1. According to the difference of elemental content, two types of Fe-9Cr alloys are respectively marked as Fe-9Cr and Fe-9Cr-C in the present work. Before irradiation, the materials were heat treated at 920 °C for 30 min, air cooled, and then tempered at 720 °C for 1 h. The average grain sizes are 75 μm and 23 μm for the Fe-9Cr and Fe-9Cr-C model alloys, respectively. Bulk samples for irradiation experiments were cut from the heat-treated steel plates by spark erosion and then ground and polished. The sample sizes of Fe-9Cr and Fe-9Cr-C are 9 mm × 6 mm × 2 mm and 8 mm × 6 mm × 2 mm, respectively.

### 2.2. Irradiation Experiments

**Figure 1:**
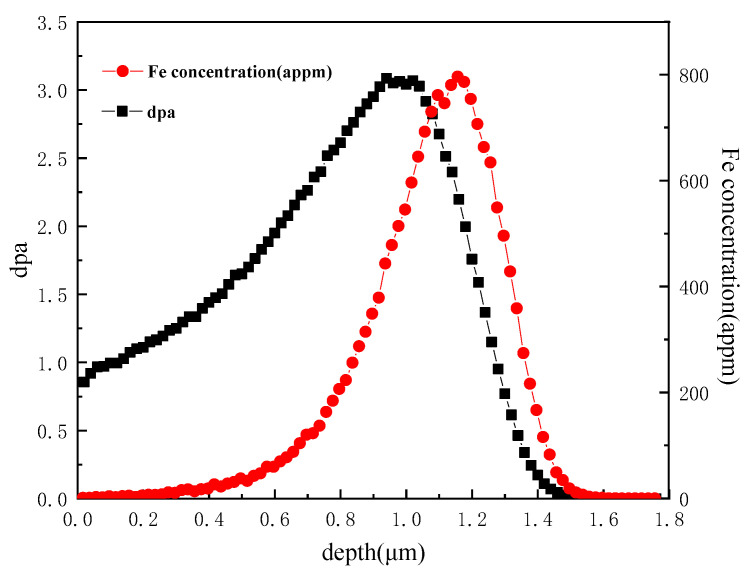
The damage profile calculated for Fe-9Cr alloy irradiated with a 3.5 MeV Fe^13+^ ion by using SRIM2013.

The irradiation experiments were performed at the 320 kV platforms for multi-discipline research with highly charged ions at the Institute of Modern Physics (IMP), Chinese Academy of Sciences. The specimens of two model alloys were irradiated simultaneously with 3.5 MeV Fe^13+^ at 450 °C or 550 °C to a dose of 3dpa at the peak damage region. The final fluence of irradiation was 3 × 10^15^ cm^−2^. Detailed irradiation conditions of these two Fe-Cr alloys are summarized in Table 2. By using SRIM2013, the displacement damage was calculated with the “Quick Calculation (K-P)” mode for the Fe ion irradiation using a displacement energy of 40 eV [23]. A typical damage profile across the Fe-9Cr sample depth is illustrated in Figure 1.

### 2.3. TEM Sample Preparation and Characterization

TEM foils for the microstructure characterization were prepared using a focused ion beam (FIB) system. Flash polishing was used to remove the FIB damage [24]. An electropolishing apparatus with an electrolytic solution of 5% perchloric acid in 95% ethanol was used to carry out flash polishing. The electric potential and temperature applied to the FIB-polished specimen were 20 V and −30 °C. The time of flash polishing was controlled between 0.1 and 0.2 s. The microstructural characterization was conducted in a JEOL JEM-F200 and Talos F200X microscope. The dislocation loops were imaged under the two-beam bright-field (BF) conditions. All size and number densities of the dislocation loops were measured from micrographs using g = 110.

## 3. Results and Discussion

### 3.1. Microstructure of Unirradiated Fe-Cr Alloys

**Figure 2:**
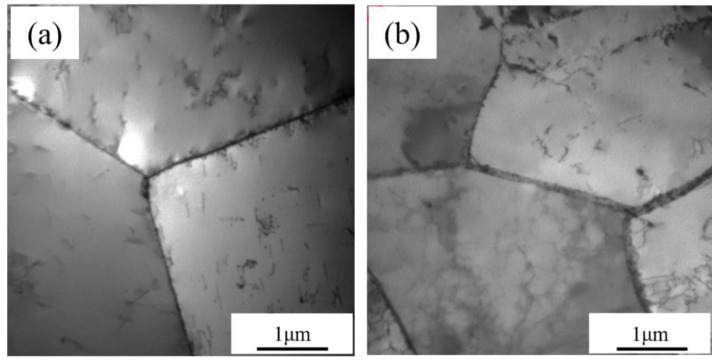
TEM bright-field micrographs of the Fe-Cr alloys before irradiation: (**a**) Fe-9Cr; (**b**) Fe-9Cr-C.

Figure 2 displays the TEM images showing the initial microstructures of these two Fe-Cr alloys. TEM observations showed that the initial microstructure of the Fe-Cr alloys is all pure ferrite. No precipitations were found on the grain boundaries or in the matrix.

### 3.2. Microstructure of Fe-Cr Alloys after Irradiation at 450 °C

**Figure 3:**
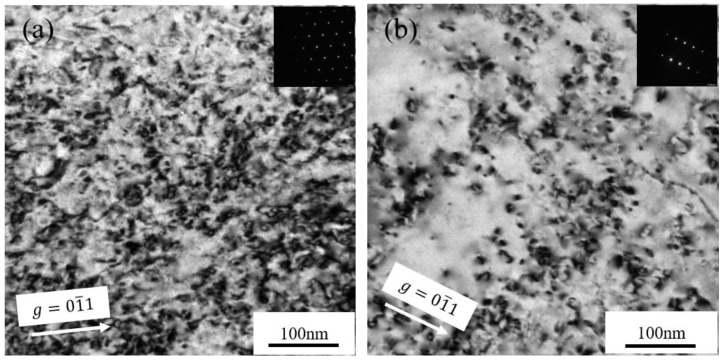
TEM bright-field micrographs showing the high-density loops in Fe-Cr alloys irradiated at 450 °C: (**a**) Fe-9Cr imaged along the (111) zone axis; (**b**) Fe-9Cr-C imaged along the (133) zone axis.

**Figure 4:**
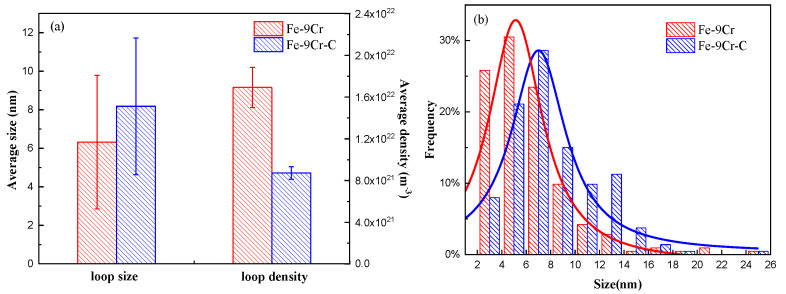
(**a**) Average size and density of the dislocation loops in the Fe-Cr alloys irradiated at 450 °C. (**b**) Size distribution of the dislocation loops in the Fe-Cr alloys irradiated at 450 °C.

Figure 3a,b displays the BF images, showing the microstructures of the Fe-9Cr and Fe-9Cr-C alloys irradiated at 450 °C, respectively. The main feature of the microstructure after irradiation is that the high-density dislocation loops were formed in the matrix. In order to compare the microstructure between the two alloys after irradiation quantitatively, the size and density of dislocation loops were measured, and the statistical results were tabulated in Table 3. The statistical region is chosen to be between the depth of 400 nm and 700 nm. Figure 4a,b shows the average size, average density, and size distribution of loops formed in the two alloys irradiated at 450 °C, respectively. The experimental results showed that the average size of loops formed in the Fe-9Cr-C alloy was larger than that in the Fe-9Cr alloy, but the density of dislocation loops in the Fe-9Cr-C alloy was lower than that in the Fe-9Cr alloy after irradiation.

In this experiment, the Fe-9Cr and Fe-9Cr-C alloys were irradiated in the same conditions, and the main difference between them was the different concentration of interstitial elements. Specifically, the C concentration of the Fe-9Cr-C alloy is four times as high as that of the Fe-9Cr alloy. Hence, it can be inferred that the difference of carbon concentration resulted in the difference of the loop size between these two Fe-Cr alloys. Atomistic simulations revealed that C atoms exhibit strong interactions with vacancies [18]. The combination of C atoms with vacancies can form thermally stable C-V complexes [25,26]. These C-V complexes are immobile and have higher energy for dissociation than the carbon atom migration energy [26]. Thus, the mobility of vacancies is decreased due to the trapping effects by C atoms. Ab initio calculations by Domain [18] demonstrated that N atoms had stronger interactions with vacancies than C atoms, and the N-V complexes were more stable than the C-V complexes. These studies have shown that interstitial elements, such as C and N, have a strong interaction with vacancies and form stable complexes with vacancies. Furthermore, the C-V complexes formed during the irradiation can trap or pin the gliding interstitial dislocation loops [27,28,29], which, in turn, affect the microstructural evolution. The experimental research of Konstantinović et al. [22] suggested that homogeneous loops with a larger size were observed in the neutron-irradiated Fe-Cr alloy, where C was uniformly dissolved in the matrix, but loops were heterogeneously distributed at grain boundaries in the Fe-Cr alloys with C segregated at boundaries and dislocations. They provided an explanation that C-V complexes formed in the Fe-Cr alloys with C uniformly dissolved in the matrix would trap and inhibit gliding interstitial dislocation loops approaching sinks, such as grain boundaries and dislocations, thereby promoting the growth of dislocation loops. It should be emphasized that the irradiation temperature (450 °C and 550 °C) in our experiment is higher than the irradiation temperature (300 °C) in the experiment of Konstantinović et al. [22]. Since the research of Konstantinović et al. [30] indicated the C-V complexes would dissociate in Fe-C alloys when the temperature was above 427 °C, it can be inferred that the stable C-V complexes would not exist in Fe-Cr-C alloy in our irradiation experiment. Therefore, the mechanism of dislocation loops trapped by C-V complexes proposed by M. J. Konstantinović et al. [22] may not explain the observed difference of dislocation loops between these two alloys after irradiation at 450 °C in our experiment.

Based on cluster dynamics, Hardouin Duparc et al. [31] deduced the stationary growth rate equation of the dislocation loops:

(1) $$\frac{dR_{l}}{dt}=\left(Z_{i}^{l}-Z_{v}^{l}\right)\left(\frac{GV_{at}}{2b\pi r_{iv}}\right)D_{v0}exp\left(-\frac{E_{v}^{m}}{2kT}\right)\;$$

where *b* is the Burgers vector of the dislocation loop; $Z_{i}^{l}$ and $Z_{v}^{l}$ are the capture efficiency of loops for the interstitial atoms and vacancies; *G* is the shear modulus; $V_{at}$ is the atomic volume; $r_{iv}$ is the recombination radius of interstitial atoms and vacancies; *k* is the Boltzmann constant; $D_{v0}$ and $E_{v}^{m}$ are the frequency factor and the migration energy for vacancy diffusion, respectively. For these two Fe-Cr alloys, there is no obvious difference in the $\left(\frac{GV_{at}}{2b\pi r_{iv}}\right)$ term; the growth rate of dislocation loops is related to the capture efficiency of loops for point defects, ($Z_{i}^{l}-Z_{v}^{l}$) and the mobility of vacancies, $D_{v0}exp\left(-\frac{E_{v}^{m}}{2kT}\right)$. As mentioned above, the mobility of vacancies can be decreased by the formation of C-V complexes, but the dissociation of C-V complexes will occur at high temperatures, which means that the difference in vacancy mobility of the two alloys can also be ignored. Therefore, the difference in the capture efficiency of loops for point defects may be the main factor affecting the loop microstructure of both Fe-Cr alloys. The dislocation loops formed in Fe-9Cr-C may have greater capture efficiency for interstitial atoms. This led to the formation of larger dislocation loops in the Fe-9Cr-C alloy.

Li et al. [32] simulated the growth kinetics of an interstitial dislocation loop by a phase-field model; their simulation results indicated that the growth rate of interstitial loops can be accelerated by the elastic interaction between the interstitial loops and the point defects. This elastic interaction originates from the local volume change and local stresses caused by the interstitial loops and point defects [32]. Molecular dynamics simulations have demonstrated that C atoms can be trapped at the periphery of the dislocation loops [33], which causes greater local strains and stresses near dislocation loops, thereby increasing the elastic interaction between the dislocation loops and the interstitial atoms. The greater elastic interaction provides dislocation loops with greater capture efficiencies for interstitial atoms, accelerating the growth of dislocation loops. This may be the reason why larger dislocation loops were formed in the Fe-9Cr-C alloy. Considering that these two alloys were irradiated simultaneously, the number of SIA atoms formed in the two alloys should be approximatively the same. Thus, when larger loops were formed in the Fe-9Cr-C alloy, the density of the loops of the Fe-9Cr-C alloy would be lower than that of the Fe-9Cr alloy.

### 3.3. Microstructure of Fe−Cr Alloys after Irradiation at 550 °C

**Figure 5:**
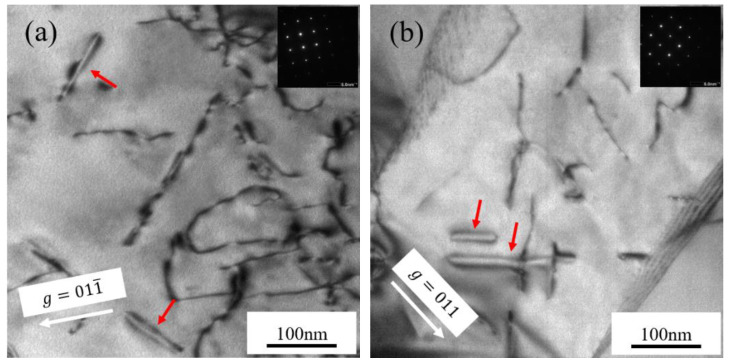
TEM bright-field micrographs showing the microstructure of the Fe-Cr alloys irradiated at 550 °C. Red arrows indicate the loops with edge-on morphology: (**a**) Fe-9Cr imaged along the (100) zone axis; (**b**) Fe-9Cr-C imaged along the (100) zone axis.

Figure 5a,b displays BF images showing the microstructures of the Fe-9Cr and Fe-9Cr-C alloys irradiated at 550 °C, respectively. Compared with alloys irradiated at 450 °C, the obvious difference in microstructural changes is that high-density dislocation loops were not observed in the Fe-Cr alloys after irradiation. Only a few loops with edge-on morphology were observed in the Fe-Cr alloys. Since the number of loops observed in the alloys irradiated at 550 °C was so low that no dislocation loops were found in most parts of the irradiation damage region, the density of the loops and the size distribution of the loops were not measured.

**Figure 6:**
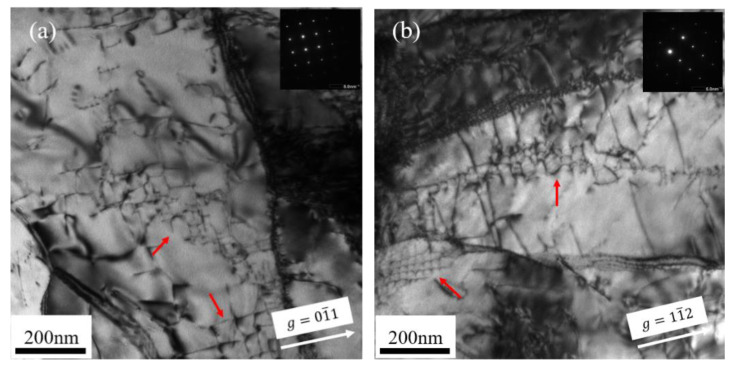
TEM bright-field micrographs showing the microstructure of Fe-Cr alloys irradiated at 550 °C; red arrows indicate the network dislocations: (**a**) Fe-9Cr imaged along the (100) zone axis; (**b**) Fe-9Cr-C imaged along the (110) zone axis.

Recent research indicated that defect evolution in the Fe and Fe-10Cr alloy with an increasing dose included the formation of black dot loops, loop coarsening, loop decoration, and the formation of network dislocations [34]. The rate of defect evolution is affected by the irradiation temperature, and the density of loops reduces rapidly at high temperatures since loops evolve into network dislocations [34,35,36]. Our experimental results indicated that the density of the dislocation loops decreases sharply in the Fe-Cr alloys irradiated at 550 °C compared to Fe-Cr alloys irradiated at 450 °C. In addition, the network dislocations were observed in both Fe-Cr alloys irradiated at 550 °C (as shown in Figure 6). The in-suit irradiation experiments indicated that the transformation of loops to network dislocations in Fe-10Cr foil irradiated at 435 °C occurred when the irradiation dose reached 18 dpa, and small loops were dominant at about 3 dpa [34]. This is consistent with our experimental results of the high-density dislocation loops observed at 3dpa when the irradiation temperature is 450 °C. At 550 °C. It can be inferred from the experimental results that the loops transformed to network dislocations before the irradiation dose reached the 3 dpa. Thus, the high-density dislocation loops were not observed in both Fe-Cr alloys.

### 3.4. The Formation of Subgrains

**Figure 7:**
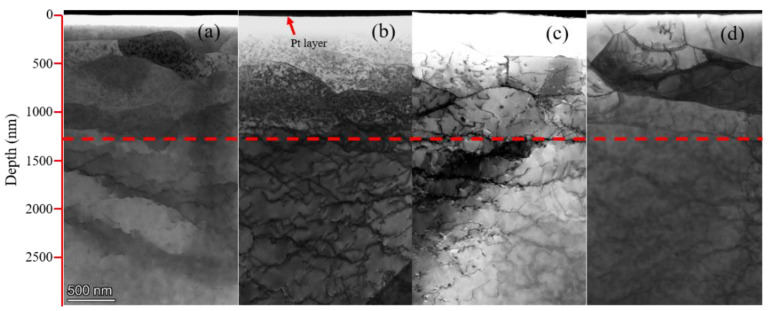
STEM bright-field micrographs showing the subgrains formed in Fe-Cr alloys; above the red lines are the irradiation damage regions: (**a**) Fe-9Cr-C irradiated at 450 °C; (**b**) Fe-9Cr irradiated at 450 °C; (**c**) Fe-9Cr irradiated at 550 °C; (**d**) Fe-9Cr-C irradiated at 550 °C.

**Figure 8:**
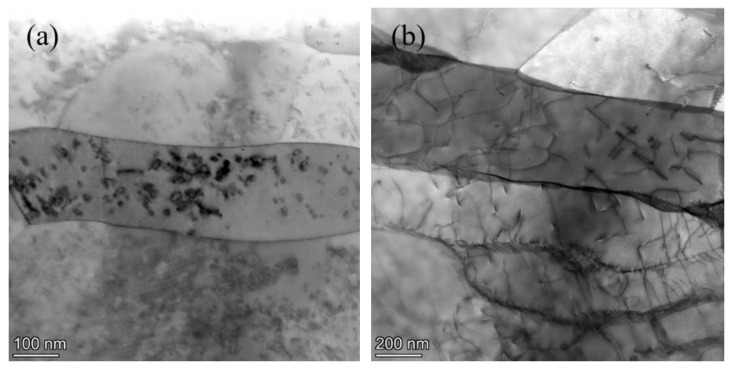
STEM-BF images showing thin, elongated subgrains: (**a**) Fe-9Cr-C irradiated at 450 °C; (**b**) Fe-9Cr-C irradiated at 550 °C.

Subgrains were observed in the irradiation damage regions of both Fe-Cr alloys not only at 450 °C but also at 550 °C. Figure 7 displays the STEM-BF images of both alloys irradiated at 450 °C and 550 °C. Above the red line are irradiation damage regions. Because the flash polishing eliminated the regions near the surface of the FIB samples, except for the Fe-9Cr-C alloy irradiated at 550 °C, there existed a blank area below the platinum layer. Obviously, subgrains exist in the irradiation damage region of both Fe-Cr alloys irradiated at 450 °C and 550 °C. Figure 8 displays the STEM-BF images showing thin, elongated subgrains at a higher magnification.

The formation of subgrains has rarely been reported for irradiation experiments on bulk metals [37,38,39]. Nesterova et al. [37] observed the formation of a subgrain structure in ion-irradiated, cold-worked, dispersion-strengthened copper alloys. Zheng et al. [38] observed the formation of subgrains near the surface in ion-irradiated bulk tungsten and attributed it to the diffusion and clustering of radiation-induced SIAs. They believed that the high concentration of SIAs developed into a high density of dislocations, and aggregation of dislocations further evolved into submicron grains [38]. For Fe-Cr alloys, only Lin et al. [39] reported the formation of subgrains in dual-ion-irradiated Fe-Cr alloys between 400–550 °C. However, Lin et al. [39] did not believe that the formation of subgrains was caused by the irradiation and attributed the formation of subgrains to the thermal expansion of samples during irradiation. In their irradiation experiments, the samples were arranged closely, and there were no gaps between them. Hence, compressive stress above the yield strength of Fe-Cr alloys caused by thermal expansion may have been induced during irradiation, which caused the subgrain formation. In other words, the formation of subgrains is an artifact caused by thermal expansion instead of a phenomenon induced by irradiation. This artifact could be eliminated by simply keeping spaces between the preirradiated samples [39].

**Figure 9:**
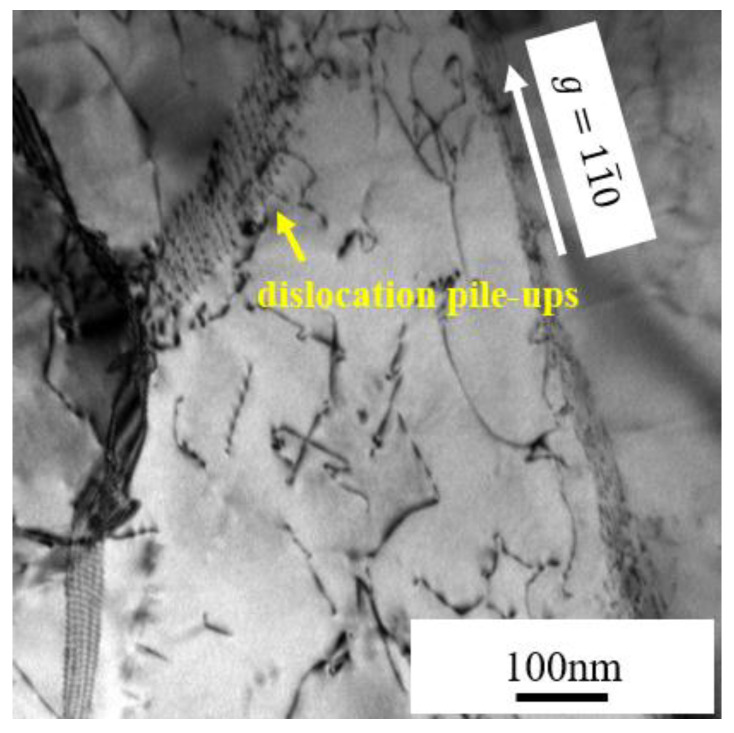
Dislocation pile-ups at a subgrain boundary in the Fe-9Cr alloy irradiated at 550 °C.

In our experiments, both Fe-Cr alloys were simultaneously irradiated. Two samples were close enough, and there was no gap between them. The irradiation temperatures in our experiment were similar to those in the experiments of Y. Lin et al. [39]. In addition, the dislocation pile-ups were found near a subboundary in the Fe-9Cr alloy irradiated at 550 °C (as shown in Figure 9). Dislocation pile-ups usually form during plastic deformation. When a series of same sign dislocations move on the same slip plane, if the leading dislocation meets a barrier, such as a grain boundary, the dislocations will then pile up behind the leading dislocation [40]. As mentioned above, Lin et al. [39] proposed that the stress induced by the thermal expansion during the irradiation process caused the formation of subgrains. The formation of dislocation pile-ups demonstrates that the stress driving dislocation slip might exist during irradiation, so our experimental results are inclined to support the view of Lin et al. [39]. The formation of subgrains is related to stress associated with the thermal expansion during irradiation. The stress caused by the thermal expansion led to the movement and interaction of the dislocations during irradiation, eventually leading to the formation of the subboundaries.

It is worth noting that the subgrains only exist in the radiation damage region in the above experiments. Attributing the formation of subgrains to the stress caused by thermal expansion cannot explain this phenomenon. Therefore, the possibility that the subgrains were caused by irradiation cannot be completely eliminated. In general, more experiments are necessary to avoid the contact of samples during the irradiation in order to further clarify the mechanism of subgrain formation during the irradiation.

## 4. Conclusions

In this study, two types of Fe-Cr model alloys with different concentration of interstitial elements were irradiated simultaneously with 3.5 MeV Fe^13+^ at 450 °C or 550 °C to a dose of 3dpa at the peak damage region. The experimental results indicated that the average size of the loops observed in Fe-9Cr-C alloy is larger than that in Fe-9Cr alloy, but the loop density in Fe-9Cr-C is lower than that in Fe-9Cr alloy after irradiation at 450 °C. The concentration of the interstitial carbon influenced the evolution of the loop microstructure in these two alloys. At 550 °C, since the loop microstructure evolved into network dislocations in Fe-Cr alloys, the density of loops decreased sharply so that no dislocation loops were found in most parts of the irradiation damage region. Subgrains were formed in the Fe-Cr alloys after irradiation; the formation mechanism of subgrains was attributed to compressive stresses induced by the thermal expansion and contact between the specimens during irradiation.

## Figures and Tables

**Table 1:** Concentrations of the composition in samples used in this study (wt.%).

Alloy	Cr	C	N	O	P	S
Fe-9Cr	8.97	0.003	0.0014	0.013	0.0074	<0.003
Fe-9Cr-C	8.90	0.012	0.0043	0.027	0.0076	<0.003

**Table 2:** The irradiation conditions of materials.

Alloy	Temperature (°C)	Dose at Peak (dpa)	Dose Rate (dpa/s)
Fe-9Cr	450	3	1.7 × 10^−4^
Fe-9Cr-C	450	3	1.7 × 10^−4^
Fe-9Cr	550	3	1.3 × 10^−4^
Fe-9Cr-C	550	3	1.3 × 10^−4^

**Table 3:** Summary of the average size and density of the dislocation loops in the Fe-Cr alloys.

Alloy	Average Size (nm) (Number of Loops Measured)	Average Density (×10^21^ m^−3^)
Fe-9Cr (at 450 °C)	6.3 ± 3.5 (213)	16.9 ± 1.9
Fe-9Cr-C (at 450 °C)	8.2 ± 3.5 (213)	8.7 ± 0.6
Fe-9Cr (at 550 °C)	32.5 ± 28.9 (15)	Not measured
Fe-9Cr-C (at 550 °C)	47.8 ± 35.0 (12)	Not measured

## Data Availability

The data presented in this study are available on request from the corresponding author.
